# Supplementary material for: Synthesis and Evaluation of Dodecaboranethiol Containing Kojic Acid (KA-BSH) as a Novel Agent for Boron Neutron Capture Therapy
Source: Cells. 2020 Jun 25;9(6):1551. doi: 10.3390/cells9061551 (PMC7349888; doi:10.3390/cells9061551)
Supplement: Supplementary file 1 [file cells-09-01551-s001.pdf]

cells MDPI

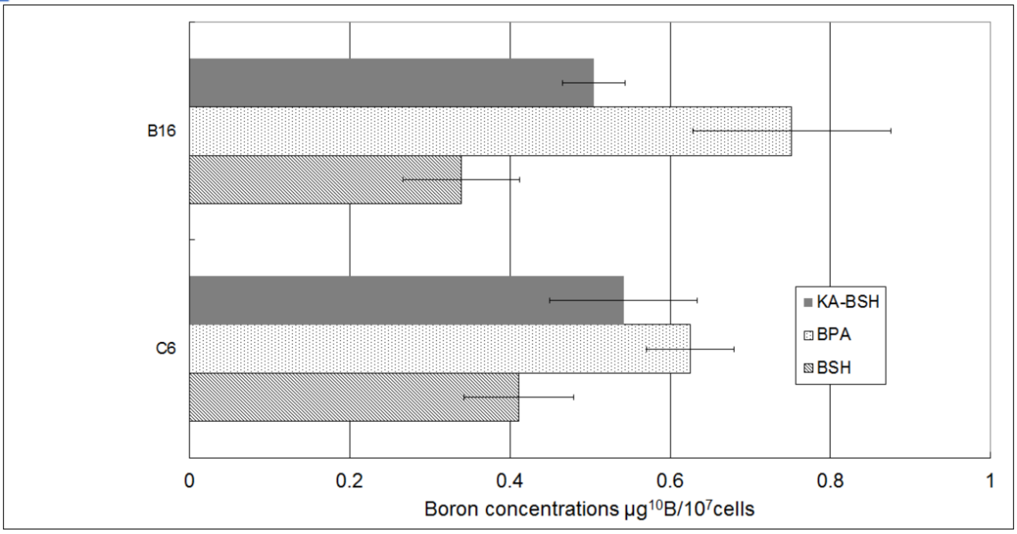

Figure S1. The boron concentrations of B16 mouse melanoma cells and C6 rat glioma cells. ^{10}B concentration of F98 rat glioma cells 24 h after incubation in DMEM, including 1 mM of each boron compound.

Table S1. Boron concentrations in F98 glioma bearing rats

Agent[a] / Route	dose (mg ^{10}B/kg)	Time (h)	n[b]	^{10}B concentrations ± SD (μg ^{10}B/g)[c]: Blood	Ipsilateral brain	Contralateral brain	Tumor	Ratios[d]: T/Bl	T/Br
KA-BSH / iv	10	1	5	4.71 ± 1.09	0.29 ± 0.07	0.24 ± 0.06	1.42 ± 0.28	0.31	5.99
		3	3	1.26 ± 0.22	0.10 ± 0.04	0.09 ± 0.01	0.85 ± 0.30	0.69	9.52
	20	1	5	8.76 ± 1.24	0.31 ± 0.10	0.24 ± 0.09	2.96 ± 0.74	0.34	13.21
		3	4	1.54 ± 0.65	0.14 ± 0.07	0.09 ± 0.05	1.47 ± 0.17	1.12	21.07
	30	1	4	14.21 ± 3.17	0.63 ± 0.09	0.45 ± 0.08	6.65 ± 0.25	0.49	15.27
		3	4	2.35 ± 1.37	0.35 ± 0.39	0.14 ± 0.06	2.91 ± 0.34	1.58	23.09
BSH / iv	30	1	4	19.17 ± 3.28	0.44 ± 0.08	0.32 ± 0.05	5.49 ± 0.25	0.29	17.61
		3	4	5.16 ± 1.89	0.36 ± 0.05	0.24 ± 0.04	3.01 ± 0.95	0.61	12.71
BPA / iv	10	1	6	6.65 ± 1.76	2.94 ± 0.67	3.19 ± 0.67	13.54 ± 4.82	2.00	4.21
		3	4	4.41 ± 1.21	3.29 ± 0.44	2.95 ± 0.72	11.05 ± 2.58	2.53	3.83

a; KA-BSH, BSH or BPA was administered by intravenous injections, administered to Fischer rats bearing intracranial implants of the F98 rat glioma. Animals were euthanized at 1 and 3 h following the injection and tissues were removed for ^{10}B determinations. b; n is the number of animals per group. c; ^{10}B concentrations were determined by ICP-AES. The mean ^{10}B values (μg ^{10}B/g weight of tissue) ± the standard deviation are shown for groups of 3–6 rats. d; T/Bl indicates the tumor to blood ratio. T/Br indicates the tumor to normal brain (contralateral brain) ratio.

Table S2. Survival times of F98 glioma bearing rats following KA-BSH

Agent/Route		Survival Time			%ILS[c]
**Group**	**n[b]**	**Mean ± SD**	**Median**	**Range**	**Median**
KA-BSH/iv[a]	5	35.4 ± 8.0	36.0	32 – 38	22.0
Irradiated Controls	6	30.2 ± 2.2	30.5	26 – 32	3.4
Untreated Controls	4	28.5 ± 3.1	29.5	24 – 31	-

a; A total of 10 mg 10B/kg of KA-BSH was administered by intravenous injection. BNCT was initiated 1 hour after the intravenous administration of KA-BSH. B; n is the number of animals per group. c; Percent increase life span (%ILS) was defined relative to the median survival times of untreated controls.
